# Taxonomic studies of pteridophytes of Ambon and Seram (Moluccas) collected on Indonesian-Japanese botanical expeditions 1983–1986. XIII. Hymenophyllaceae

**DOI:** 10.3897/phytokeys.119.33565

**Published:** 2019-04-01

**Authors:** Kunio Iwatsuki, Atsushi Ebihara, Masahiro Kato

**Affiliations:** 1 815-29 Kamoshida, Aoba-ku, Yokohama 227-0033, Japan Unaffiliated Yokohama Japan; 2 Department of Botany, National Museum of Nature and Science, 4-1-1 Amakubo, Tsukuba 305-0005, Japan Department of Botany, National Museum of Nature and Science Tsukuba Japan

**Keywords:** Ambon, filmy ferns, Hymenophyllaceae, Malesia, pteridophyte flora, Seram

## Abstract

Identifications are given for 713 specimens of Hymenophyllaceae collected on Ambon and Seram islands, the Moluccas, Indonesia, during 1983–86. The collection is composed of forty-seven species and one variety belonging to seven genera. The dataset is deposited in GBIF and available at https://www.gbif.jp/ipt/resource?r=seram_hymen.

## Introduction

The flora of Seram and Ambon islands, the Moluccas, covering bryophytes, pteridophytes and seed plants, was investigated during field expeditions in 1983, 1984–85 and 1986. As the Moluccan islands, in particular Seram, have been explored very sparsely, the expeditions aimed to make general collections of the land plants in the area. More than 11,000 field numbers of vascular plant and 5,000 bryophyte specimens were collected, mainly from east, central and west Seram (Kato 1990a).

The pteridophyte flora of Seram and Ambon was revised by Kato (1990a), who has continuously contributed to this topic, based on his taxonomic studies in identifying our collections. Kato (1990a) provided an overview of the fern flora of Seram and implied that well over 700 species of pteridophytes occur on the island, based on the collection of nearly 700 species on only three explorations. The collection lists of pteridophytes, excluding Hymenophyllaceae, were already published by M. Kato and his collaborators (Kato 1988, 1989a, 1989b, 1990b, 1992, 1994, 1996, 1997, 2007, Kato and Kramer 1990, Kato and Parris 1992, Kato and Price 1990).

## Description

The GBIF dataset is a list of specimens of Hymenophyllaceae collected in Seram and Ambon from 1983 to 1986. The first two sets of specimens of this family are kept in TI and BO; the third more or less incomplete set will be in L, with a few more duplicates to be distributed to other herbaria. In total, 47 species of the Hymenophyllaceae are recorded.

Seram has an area of about 17,000 km^2^ with many mountain peaks reaching 2,000 to 3,000 m elevation, the highest being 3,019 m. When the collections were made in 1980s, most mountainous areas were still natural and undeveloped, covered mostly with primary forests. Mountains over 1500 m elevation are in a cloud zone of mossy forests where filmy ferns prefer to grow. For the species enumerated here, the habitat of each species is summarised and edited from the field notes on the collection labels. There are widespread calcareous areas on Seram Island and most collections are from such areas. Ambon is a much smaller island, located southwest of Seram. The flora of Ambon had been relatively better known than Seram’s because of the epoch-making pre-Linnean work of G. E. Rumphius’ Herbarium Amboinense. However, the island is now well populated and has been deforested. A small number of Hymenophyllaceae were collected on Ambon. On Seram Island, no particular species of filmy fern necessarily grow in limestone areas. For the epiphytic species, calcareous habitats appear to be of less concern. The epipetric species cited in the following list usually grow on very wet, often moss-covered limestone as facultative calcareous species.

### Data published through GBIF

https://www.gbif.jp/ipt/resource?r=seram_hymen

### Geographic coverage

Ambon Island and Seram Island (the Moluccas), Indonesia.

### Taxonomic coverage

Hymenophyllaceae.

### Study area

Ambon and Seram islands, the Moluccas, Indonesia. The collection route map for the 1983 trip is given in Kato et al. (1984: 150–151). The collection sites on Seram Island are given in Kato (1990a).

### Sampling methods

Pteridophytes specimens, including those of Hymenophyllaceae, were collected in Seram and Ambon islands on the expeditions 1983–1986.

The pteridophyte flora of Ambon and Seram (Ceram) was comprehensively explored in the 1980s and was studied by M. Kato and his colleagues during 1985 and 2007. Most of the pteridophyte collections have already been studied, but the specimens of Hymenophyllaceae remained unprocessed. After identification of the specimens by the authors, following the classification system by Ebihara et al. (2006), seven genera and 47 species are here recorded. The diversity of species is equivalent to nearly half of all species in Malesia, where 108 species have now been identified and recorded (Iwatsuki and Ebihara in prep.).

The field research was organised as a joint survey by the Botanical Gardens, the University of Tokyo and Herbarium Bogoriense, LIPI. Along with the work on the Hymenophyllaceae for Flora Malesiana, this taxonomically interesting family has been revised and the taxonomy of the species has been determined, including the identification of the collections cited here. A modern system, including information based on molecular systematics, was proposed by Ebihara et al. (2006), although further study is continuing. This list of Ambon and Seram species is arranged according to the system proposed there, except for the order of the infrageneric taxa.

### Key to the subgenera and species

See Ebihara et al. (2006) for key to the genera.

### *Hymenophyllum* Sm.

**Table:** 

1	Stellate hairs present on fronds, and/or fronds dichotomously divided (subgen. Sphaerocionium)	**2**
–	Stellate hairs absent on fronds and/or fronds not dichotomously divided	**4**
2	Fronds nearly glabrous or with occasional soft hairs at margin	**2. *H.nitidulum* (Blume) Ebihara & K. Iwats**
–	Obvious hairs present on fronds	**3**
3	Fronds with soft stellate hairs on costae and margin of segment	**3. *H.palmatifidum* (Bosch) Ebihara & K. Iwats**
–	Fronds setose, at margin of segments, hairs dark brownish	**4. *H.digitatum* (Müll. Berol.) Ebihara & K. Iwats**
4	Fronds glaucous or covered with whitish multicellular hairs (subgen. Pleuromanes)	**1. *H.pallidum* (Blume) Ebihara & K. Iwats**
–	Fronds neither glaucous nor covered with whitish multicellular hairs	**5**
5	Rhizome more than 0.4 mm in diameter, nearly glabrous or with scattered pale hairs (subgen. Globosa)	**6**
–	Rhizome filiform, less than 0.4 mm in diameter, with scattered short brown hairs	**16**
6	Receptacles filiform to columnar, involucres triangular to subdeltoid, longer than wide or rarely reniform, nearly as long as wide	**7**
–	Receptacles capitate, involucre distinctly broader than long	**12**
7	Wings of axes flat or undulate, lips of involucre entire or at most crenulate (or toothed in *H.productum*)	**8**
–	Wings of axes and ultimate segments distinctly crisped at margin	**9**
8	Lips of involucre entire or at most crenulate	**5. *H.angulosum*H. Christ**
–	Lips of involucre toothed; segments often laxly placed with some irregularly elongated ones	**6. *H.productum* Kunze**
9	Lips of involucre entire to crenate	**10**
–	Lips of involucres toothed to fimbriate	**11**
10	Lamina of fronds > 8cm; wings of axes distinctly crisped	**7. *H.reinwardtii* Bosch**
–	Lamina of fronds < 8(-10) cm; wings exceedingly crisped, margin of narrower segments appearing toothed	**8. *H.thuidium* Harr.**
11	Margin of segments flat or undulate, lips of involucres toothed	**9. *H.javanicum* Spr.**
–	Margin of segments distinctly crisped, lips of involucre fimbriate	**10. *H.fimbriatum* J. Sm.**
12	Margin of wings and ultimate segments flat	**13**
–	Margin of wings and ultimate segments more or less crisped	**11. *H.badium* Hook. & Grev.**
13	Fronds in general ovate to oblong-ovate	**14**
–	Fronds narrowly lanceolate	**13. *H.longifolium* Alderw.**
14	Head of receptacles widened	**15**
–	Head of receptacles globose	**14. *H.imbricatum* Blume**
15	Involucres crenate; wings usually narrower than or the same as segments	**11. *H.badium* Hook. & Grev.**
–	Involucres entire; wings of rachis broad, often > 1 mm wide, flat and entire	**12. *H.junghunii* Bosch**
16	Margin of segments toothed (subgen. Hymenophyllum)	**17**
–	Margin of segments entire	**24**
17	Rachis terete basally, wings of upper part of rachis narrow and flat	**18**
–	Rachis winged throughout, wings more or less crisped, lips of involucre entire or serrate	**21**
18	Mature fronds normally > 3 cm long	**19**
–	Mature fronds < 3 cm long	**19. *H.blandum* Racib.**
19	Fronds normally > 6 cm long, more or less lax; sori < 4 mm long, not blackish	**20**
–	Fronds < 6 cm long, more or less compact; sori about 4 mm long, blackish	**24. *H.melanosorum* (Copel.) C. V. Morton**
20	Segments about 7–10 mm broad, sori < 3 mm long, fronds not dark when dried	**16. *H.serrulatum* (C. Presl) C. Chr.**
–	Segments about 7 mm broad, sori 3–4 mm long, dark brownish when dried	**17. *H.klabatense*H. Christ**
21	Wings not toothed	**22**
–	Wings toothed	**23**
22	Fronds not black when dried, ultimate segments about 1 mm broad, dentation regular, with few cells	**18. *H.holochilum* (Bosch) C. Chr.**
–	Fronds blackish when dried, ultimate segments 0.3–0.7 mm broad, dentation sharp and distinct, with several rows of cells	**23. *H.rosenstockii* Brause**
23	Wings more or less crisped	**20. *H.denticulatum* Sw.**
–	Wings plane	**21. *H.acanthoides* (Bosch) Rosenst.**
24	Laminar cell walls thin and straight; receptacles included (subgen. Mecodium)	**15. *H.polyanthos* (Sw.) Sw., s.l.**
–	Laminar cell walls more or less thick; receptacle extruded beyond lips of involucres (subgen. Hymenophyllum)	**25**
25	Fronds usually > 10 cm long, axes of fronds sparsely hairy	**22. *H.* sp. 1**
–	Fronds usually < 7 cm long, axes of fronds rather densely hairy	**25. *H.pachydermicum* Cesati**

### *Didymoglossum* Desv.

**Table:** 

1	1. Submarginal false veinlets absent (subgen. Didymoglossum)	**2**
–	1. Submarginal false veinlets present (subgen. Microgonium)	**28. *D.bimarginatum* (Bosch) Ebihara & K. Iwats.**
2	Fronds simple, stipitate, attached at base (or not peltate), the lower surface glabrescent	**26. *D.motleyi* (Bosch) Ebihara & K. Iwats.^1^**
–	Fronds sessile, circular and subentire, peltate, lower surface with hairs along veins	**27. *D.tahitense* (Nadeaud) Ebihara & K. Iwats.**

### *Crepidomanes* (C. Presl) C. Presl

**Table:** 

1	Rhizome slender, < 2 mm in diameter, long creeping, fronds < 10 cm long (subgen. Crepidomanes)	**2**
–	Rhizome thick, > 2 mm in diameter, erect or creeping, fronds > 10 cm long (subgen. Nesopteris)	**10**
2	False veinlets present, if absent, without differentiated marginal cells and gemmae (sect. Crepidomanes)	**3**
–	Segments without false veinlets	**8**
3	Mature fronds usually > 5 cm long, texture more or less firm	**4**
–	Mature fronds smaller, usually < 4 cm long, texture soft and delicate	**6**
4	Submarginal veinlets continuous without any interruption, the additional oblique striae none or few	**29. *C.bipunctatum* (Poir.) Copel.**
–	Submarginal veinlets, if any, not continuous, oblique striae present	**5**
5	Submarginal veinlets continuous but interrupted	**29. *C.bipunctatum* (Poir.) Copel.**
–	Submarginal veinlets obsolete, with abundant oblique striae	**30. *C.latealatum* (Bosch) Copel.**
6	Submarginal veinlets present, continuous or interrupted	**7**
–	Submarginal veinlets obsolete; fronds simple to pinnately compound	**33. *C.pervenulosum* (Alderw.) Copel.**
7	Two rows of normal cells present outside submarginal strands	**31. *C.brevipes* (C. Presl) Copel.**
–	Only one row of normal cells present outside submarginal strands	**32. *C.kurzii* (Bedd.) Tagawa & K. Iwats.**
8	Marginal cells not differentiated	**9**
–	One or two marginal rows of cells differentiated from others (sect. Crepidium)	**36. *C.humile* (G. Forst.) Bosch**
9	Fronds sessile to subsessile; stipe never gemmiferous (sect. Crepidomanes)	**34. *C.vitiense* (Baker) Bostock**
–	Fronds never sessile to subsessile; stipe always distinct, wingless and often gemmiferous (sect. Gonocormus)	**35. *C.minutum* (Blume) K. Iwats.**
10	Without abortive fronds	**37. *C.intermedium* (Copel.) Ebihara & K. Iwats.**
–	With abortive fronds at base of normal fronds	**38. *C.aphlebioides* (H. Christ) I. M. Turner**

### *Vandenboschia* Copel.

**Table:** 

1	Terrestrial or saxicolous plants or at most on base of tree trunks; fronds decompound, at least tripinnate (subgen. Vandenboschia)	**39. *V.maxima* (Blume) Copel.**
–	Scandent plants, usually on branches of trees; fronds lanceolate to narrowly so, simply pinnate (subgen. Lacosteopsis)	**40. *V.auriculata* (Blume) Copel.**

### *Abrodictyum* C. Presl

**Table:** 

1	Rhizome short-creeping; laminar cells up to 1 mm long, tetragonal to elongate, variously arranged (subgen. Abrodictyum)	**2**
–	Rhizome erect or ascending; laminar cells up to 0.2 mm long, almost all tetragonal, close to each other (subgen. Pachychaetum)	**44. *A.obscurum* (Blume) Ebihara & K. Iwats.**
2	Ultimate segments narrow, setaceous, in several planes in cubic arrangement, laminar cells obsolete or only in one row at each side of costa	**42. *A.pluma* (Hook.) Ebihara & K. Iwats.**
–	Ultimate segments narrow but not setaceous, arranged in one plane, laminar cells in 2–4 rows at each side of costa	**3**
3	Terrestrial ferns with erect or short ascending rhizome; fronds < 10 cm long; stipes rather sparsely hairy; ultimate segments narrow, with 2–4 rows of cells on each side of costa	**43. *A.idoneum* (C. V. Morton) Ebihara & K. Iwats.**
–	Epiphytic or epipetric ferns with creeping rhizome; fronds usually 20–50 cm long; stipes with dense bristles throughout, bristle > 8 mm in length; ultimate segments various, forming more or less cubic construction of fronds, broader, usually with 3–6 larger, elongate cells rather obliquely arranged on each side of costa	**41. *A.schlechteri* (Brause) Ebihara & K. Iwats.**

### *Cephalomanes* C.Presl

**Table:** 

1	Mouth of involucre dilated	**45. *C.atrovirens* C. Presl**
–	Mouth of involucre truncate or hardly dilated	**2**
2	Sori on acroscopic margin of pinnae, not on basiscopic margin	**46a. C.javanicum(Blume)C. Preslvar.javanicum**
–	Sori on distal portion of pinnae and distributed towards acroscopic margin or sometimes on basiscopic margin	**46b. C.javanicum(Blume)C. Preslvar.asplenioides (C. Presl) K. Iwats.**

### *Callistopteris* Copel. (a single species in the area)

**47. *C.apiifolia* (C. Presl) Copel.**

### Dataset description

**Object name**: A Specimen List of Hymenophyllaceae of Seram and Ambon collected on Indonesian-Japanese botanical expeditions 1983–1986

**Character encoding**: UTF-8

**Metadata Language**: English

**Resource Language**: English

**Type**: Occurrence

**Subtype**: Specimen

**Data License**: Creative Commons Attribution (CC-BY) 4.0

**Thesaurus**: GBIF Dataset Subtype Vocabulary: https://rs.gbif.org/vocabulary/gbif/dataset_subtype.xml

## Footnotes

Senterre et al. (2017) proposed a hypothesis that D.beccarianum Senterre & Rouhan can be segregated from D.motleyi sensu stricto (type collection only), based on morphological characters. Here, we accept D.motleyi in a broad sense, including D.beccarianum.
